# P-1961. Epidemiologic analysis of an alpha variant COVID-19 nosocomial cluster that occurred after completed vaccination of healthcare workers

**DOI:** 10.1093/ofid/ofae631.2120

**Published:** 2025-01-29

**Authors:** Hideki Kawamura, Koji Nakano, Norihisa Hanada, Koji Sameshima

**Affiliations:** Kagoshima University, Kagoshima, Kagoshima, Japan; Izumi General Medical Center, Izumi, Kagoshima, Japan; Izumi General Medical Center, Izumi, Kagoshima, Japan; Izumi General Medical Center, Izumi, Kagoshima, Japan

## Abstract

**Background:**

In a field where few reports have been made on the efficacy of SARS-CoV-2 vaccines in healthcare settings. We experienced an outbreak of an alpha variant in a medical institution cluster after healthcare workers were vaccinated, providing a novel perspective on the topic.

**Figure 1.**
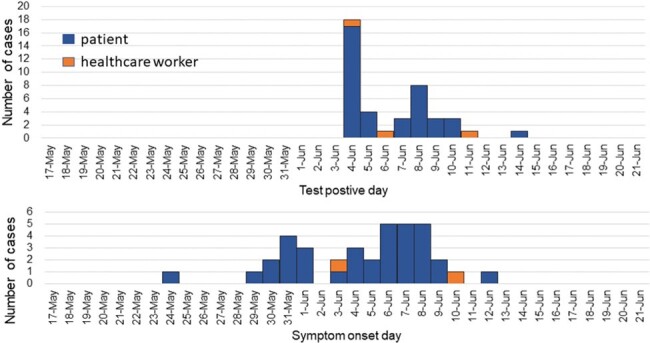
Epidemic curve based on the day of positive SARS-CoV-2 PCR test (A) or on the symptom onset day (B) during this COVID-19 outbreak

The index case was a patient admitted to the hospital on 24 May. The last positive case was detected ten days after the first detection, but 42 patients, including five asymptomatics and three healthcare workers, including an asymptomatic, were infected.

**Methods:**

We conducted descriptive epidemiology from case detection in the cluster of medical institutions with the alpha variant in June 2021. We also compared the relationship between vaccination status and infection using the Fisher exact test for healthcare workers in the same period.

**Figure 2.**
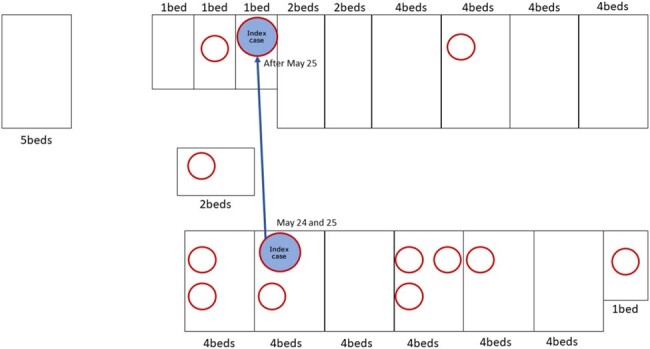
Diagram of hospital room layouts for patients infected in the first wave (through June 1)

We confirmed infected inpatients in a wide range of hospital rooms.

**Results:**

We identified one healthcare worker as a first test-positive case on 4 Jun 2021, and 21 positive patients were identified by screening on the day of detection and the following day. Based on the descriptive epidemiological analysis, the index case was a patient admitted to the hospital on 24 May with pneumonia treated with NPPV in an independently air-conditioned negative pressure room from the third day of admission. The last positive case was detected 10 days after first detection, but 42 patients and three healthcare workers were infected (Figure 1).

Forty-two of 88 hospitalized patients (53.7%) and 3 of 38 healthcare workers (7.9%) were infected. There were no cases of infection among the 30 healthcare workers who had completed the 2-dose vaccination, and the infection rate was significantly lower than that of the staff members who had not completed the vaccination (0% (0/30) vs. 37.5% (3/8), p=0.008). No hospitalized patients had completed the 2-dose vaccination, including infected and uninfected cases. Aerosol transmission from initial cases, the patient-to-patient droplet route, and the hospital ward contact route were considered factors in the spread of the disease (Figure 2 and Figure 3).

**Figure 3.**
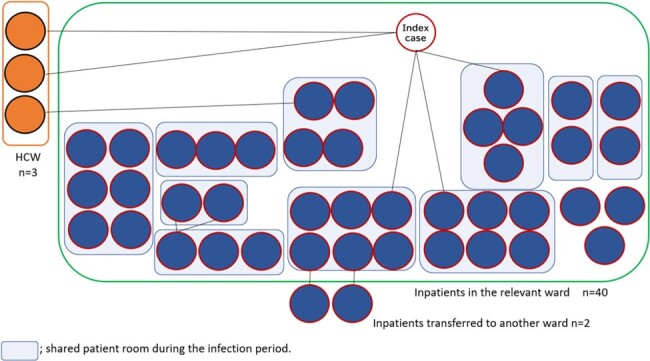
Links within the cluster

Aerosol transmission from initial cases, the patient-to-patient droplet route, and the hospital ward contact route were considered factors in the spread of the disease.

**Conclusion:**

Our study not only underscores the importance of strict adherence to standard precautions, early detection of infected cases, and implementation of route-specific prophylaxis but also highlights the crucial role of vaccination in preventing the outbreak and spread of COVID-19 clusters in medical institutions.

**Disclosures:**

All Authors: No reported disclosures

